# Ethnic Disparities in the Management of Inflammatory Bowel Disease in Israel and Impact on Outcomes

**DOI:** 10.1093/crocol/otaf025

**Published:** 2025-03-31

**Authors:** Elad Boaz, Oren Ledder, Ariella Bar-Gil Shitrit, Amir Dagan, Michael R Freund, Benjamin Koslowsky, Rona Lujan, Shira Greenfeld, Revital Kariv, Yiska Loewenberg Weisband, Natan Lederman, Eran Matz, Iris Dotan, Dan Turner, Shlomo Yellinek

**Affiliations:** Department of General Surgery, The Eisenberg R&D Authority, Shaare Zedek Medical Center, Jerusalem, Israel; Faculty of Medicine, The Hebrew University of Jerusalem, Jerusalem, Israel; Juliet Keidan Institute of Pediatric Gastroenterology and Nutrition, Shaare Zedek Medical Center, Jerusalem, Israel; Faculty of Medicine, The Hebrew University of Jerusalem, Jerusalem, Israel; Digestive Diseases Institute, Shaare Zedek Medical Center, Jerusalem, Israel; Faculty of Medicine, The Hebrew University of Jerusalem, Jerusalem, Israel; Department of General Surgery, The Eisenberg R&D Authority, Shaare Zedek Medical Center, Jerusalem, Israel; Faculty of Medicine, The Hebrew University of Jerusalem, Jerusalem, Israel; Department of General Surgery, The Eisenberg R&D Authority, Shaare Zedek Medical Center, Jerusalem, Israel; Faculty of Medicine, The Hebrew University of Jerusalem, Jerusalem, Israel; Digestive Diseases Institute, Shaare Zedek Medical Center, Jerusalem, Israel; Faculty of Medicine, The Hebrew University of Jerusalem, Jerusalem, Israel; Juliet Keidan Institute of Pediatric Gastroenterology and Nutrition, Shaare Zedek Medical Center, Jerusalem, Israel; Maccabi Health Services, The Sackler Faculty of Medicine, Tel Aviv University, Tel-Aviv, IsraelTel-Aviv, Israel; The Sackler Faculty of Medicine, Tel Aviv University, Tel-Aviv, Israel; Maccabi Health Services, The Sackler Faculty of Medicine, Tel Aviv University, Tel-Aviv, IsraelTel-Aviv, Israel; The Sackler Faculty of Medicine, Tel Aviv University, Tel-Aviv, Israel; Clalit Health Services, Clalit Research Institute, Tel-Aviv, Israel; Meuhedet Health Services, Tel-Aviv, Israel; Leumit Health Services, Tel-Aviv, Israel; Faculty of Medicine, The Hebrew University of Jerusalem, Jerusalem, Israel; Division of Gastroenterology, Rabin Medical Center, Petah Tikva, Israel; Juliet Keidan Institute of Pediatric Gastroenterology and Nutrition, Shaare Zedek Medical Center, Jerusalem, Israel; Faculty of Medicine, The Hebrew University of Jerusalem, Jerusalem, Israel; Department of General Surgery, The Eisenberg R&D Authority, Shaare Zedek Medical Center, Jerusalem, Israel; Faculty of Medicine, The Hebrew University of Jerusalem, Jerusalem, Israel

**Keywords:** inflammatory bowel disease, ethnicity, disparities

## Abstract

**Background:**

In this nationwide study, we aimed to explore healthcare services utilization, medical management, and disease outcomes of inflammatory bowel diseases (IBD) across the 2 major ethnic groups in Israel.

**Methods:**

We utilized a cohort including all patients diagnosed with IBD in Israel since 2005. The primary outcome was steroid dependency, with secondary outcomes including use of biologics, time to surgery, and hospitalizations. Outcomes were controlled for possible inherent differences in disease course and phenotype.

**Results:**

Of the 32 491 included patients, 18 252 (56%) had Crohn’s disease (CD) and 14 239 (44%) had ulcerative colitis (UC); 10% were Arabs and 90% were Jews. Jewish ethnicity was associated with lower rates of steroid dependency compared to Arab ethnicity in both CD (HR = 0.7 [95% CI, 0.6-0.8]) and UC (HR = 0.7 [95% CI, 0.6-0.8]). The risk of IBD-related surgery in CD was higher in the Arab group at both 3 and 5 years (13% vs. 10%, 16% vs 14%, respectively, *P* = .005). Arabs had more frequent IBD-related hospitalizations than Jews at 5 years (28% vs. 19% with at least 2 hospitalizations, *P* < .001). In contrast, Jewish ethnicity was associated with more frequent use of biologics during the first year from diagnosis in patients with CD (HR = 1.3 [95% CI, 1.1-1.6]) but not with UC.

**Conclusions:**

Arab ethnicity is associated with higher rates of hospitalizations, steroid dependency, and surgeries, and, on the other side, with lower utilization of biologics. Healthcare practitioners and policymakers should address potential cultural and systemic barriers in healthcare delivery in order to improve care across all populations.

## Introduction

The management of inflammatory bowel diseases (IBD) has become a highly specialized field of medicine, with care often delivered in centralized, multidisciplinary centers, which may not be accessible to all patients. Differences in health care services utilization and disease outcomes among minorities have been described in various illnesses.^1,2^ Several studies indicate that Black IBD patients may be less likely to receive biologics or to be steroid-free compared to Caucasians,^3,4^ and have more frequent emergency room visits and IBD-related hospitalizations, and higher IBD-related mortality.^5,6^ In Canada, Indigenous patients exhibit a higher hazard rate for IBD-specific and related hospital admissions, alongside fewer prescription claims for IBD medications compared to the general population.^7^ Similarly, a nationwide study in Israel by Ledder et al. identified that residence in peripheral regions and lower socioeconomic status (SES) were independently linked to higher rates of steroid dependency and surgeries among IBD patients.^8^ Additionally, Rinawi et al. found that Israeli Arab children with IBD presented with more severe disease at diagnosis than Jewish children, a difference not attributed to delayed diagnosis.^9^ These disparities highlight the challenge of determining whether such differences stem from genetic and phenotypic variations in disease characteristics or from inequities in access to healthcare among underserved populations.

In Israel, the 2 major ethnic groups are Jews and Arabs, comprising 74% and 21% of the population, respectively. All citizens have mandatory health insurance, provided by 4 health maintenance organizations (HMOs), which provide a considerable basket of health services, including almost all newly approved biologics. Private medical insurance may be purchased to cover medications and other health services outside the basket. According to the central bureau of statistics, 86% of the Jewish population has supplementary private insurance compared with only 38% among the Arab population. The socioeconomic status of Israeli Arabs is lower than that of the Jewish population. For instance, in the year 2015, the mean net monthly income for Arabs was 58% of that of non-Arabs^10^ and the proportion of Arabs with higher education remains lower than that of non-Arabs.^11,12^ We have previously found that IBD is less prevalent in the Arab population in Israel reaching 0.24% in 2018 vs 0.59% in the Jewish population, but the prevalence is rapidly increasing in both populations.^13^ Despite these differences, data on IBD management across ethnic groups in Israel are limited and based on selected populations.^9,14,15^

In this nationwide study, we aimed to explore and compare the medical management and disease outcomes between Jewish and Arab IBD patients, the 2 major ethnic groups in Israel, while controlling for disease characteristics, demographics, and socioeconomic status (SES).

## Methods

This epi-IIRN (epidemiology group of the Israeli IBD Research Nucleus) cohort includes patient data of all 4 HMOs in Israel, representing 98% of the population. Since 2000-2003 (depending on the HMO), all health care visits, laboratory test results, medications, and other ambulatory health services have been fully computerized and stored on 4 central servers. We utilized previously validated HMO-specific algorithms to identify IBD patients within the administrative databases (99% specificity, 89% sensitivity, 92% positive predictive value, 99% negative predictive value).^16^ Data obtained from the HMOs were linked with the Ministry of Health’s national registries to obtain prospective validated records on surgeries and admissions (using deterministic linking). The incidence cohort, utilized in this study, includes all patients diagnosed with IBD since 2005 to allow an adequate “look back” period of 2-5 “clean” years without any IBD-related ICD9 codes or medications in the electronic record, depending on the HMO. The longer the preceding period without an indication of IBD in the database (ie, “look-back” period), the higher the likelihood that the first documented code or IBD-related medication in the database indeed reflects the true diagnosis date. All patients were followed from diagnosis until death or June 30, 2020. SES was determined using a standardized system provided by Points Location Intelligence^T^, which is based on Israel Central Bureau of Statistics socioeconomic data with additional variables such as finances, level of formal education, real estate, local facilities, and other factors, grouped into 4 categories from 1 (lowest) to 4 (highest). Geographic data of residence was determined by postcode and defined as central, representing more densely populated urban and suburban regions, and northern and southern regions, representing more peripheral and rural populations. Central regions have ready access to several tertiary referral centers, as opposed to northern and southern regions with more restricted access to, at most, secondary referral centers. Inflammatory bowel disease patients were clustered into disease severity groups based on the laboratory results closest to IBD diagnosis date by hierarchical clustering, as previously described in detail.^17^

The primary outcome was steroid dependency, defined as cumulative use of systemic corticosteroids for more than 90 days in a year. This outcome was selected since steroid dependency is considered as a marker of suboptimal management and uncontrolled disease.^18^ The fact that medications are provided almost free of charge to all citizens in Israel ensures that steroid dependency could be avoided even in low SES populations. Other outcomes, such as use of biologics, hospitalizations, and surgery rates, may be impacted by contrasting directions of effect, reflecting severity of the disease due to cross-ethnic differences in disease characteristics, poor disease control in an underprivileged population, or easier access to the medical system by the more affluent population. Nonetheless, we included time to IBD-related surgery, use of biologics during the first year from diagnosis (consistent with recent studies that suggest that early use of biologic improve disease outcome^19^), and IBD-related hospitalizations as secondary outcomes, reported with an attempt to control for potential confounding factors.

### Statistical analysis

Descriptive data are presented as mean ± standard deviation or median (interquartile range [IQR]) and compared by Student’s *t*-test, Wilcoxon rank sum test, and *χ*^2^, as appropriate. Cox proportional-hazards models explored the association between ethnicity and specified disease outcomes adjusted for gender, age at diagnosis, health care visits, and disease severity as possible confounders. The possibility of effect modification by ethnicity and SES was determined by adding interaction terms to the models. Time-to-event was calculated with Kaplan–Meier curves and Log-rank test was used to assess the differences between the ethnic groups. *P* < .05 was taken as the significance threshold. This study was approved by the ethics committee of our institution.

## Results

A total of 32 491 patients with IBD were diagnosed after 2005 and included in the analysis: 18 252 (56%) with Crohn’s disease (CD) and 14 239 (44%) with ulcerative colitis (UC). Patients were further classified according to ethnicity as Arabs (3283, 10%) and Jews (29 208, 90%; Table 1). The median follow-up of patients with IBD was slightly shorter among Arabs (7.0 [IQR 2.7-11] years) than Jews (7.4 [IQR 3.0-12] years), and thus we focused on survival analyses and rates at predefined early time points. Arabs were generally diagnosed with IBD at a younger age than Jews, and, therefore, age was included in all multivariable models as a potential confounding variable along with age at diagnosis, gender, disease severity, SES, health care visits, and smoking status.

**Table 1.** Baseline characteristics of the included patients by ethnicity.

	Arabs (*n* = 3283)	Jews (*n* = 29 208)	*P*-value
**Gender** (male)	1797 (55%)	14 634 (50%)	<.001
**Age at diagnosis** (years) Crohn’s disease Ulcerative colitis	32 ± 16 35 ± 16	34 ± 18 39 ± 19	<.001 <.001
**Follow-up** (years) Crohn’s disease Ulcerative colitis	6.8 ± 4.3 7.3 ± 4.3	7.3 ± 4.3 7.6 ± 4.4	<.001 .015
**Geographic district**			<.001
Central	1363 (42%)	23 012 (79%)	
Southern	130 (4%)	3557 (12%)	
Northern	1779 (54%)	2623 (9%)	
**Socioeconomic score**			<.001
1 (lowest)	2560 (85%)	4779 (17%)	
2	412 (14%)	10 017 (35%)	
3	33 (1.1%)	9979 (35%)	
4 (Highest)	4 (0.1%)	3750 (13%)	
**Disease Severity**			<.001
Mild	1543 (47%)	14 693 (50%)	
Moderate	1231 (37%)	11 117 (38%)	
Severe	509 (15%)	3397 (13%)	
**Smoking** Crohn’s disease Ulcerative colitis	594 (35%) 344 (22%)	5346 (32%) 3528 (28%)	.021 <.001

Data are presented as counts (%), mean ± SD or median (IQR) as appropriate.

**Figure 1.**
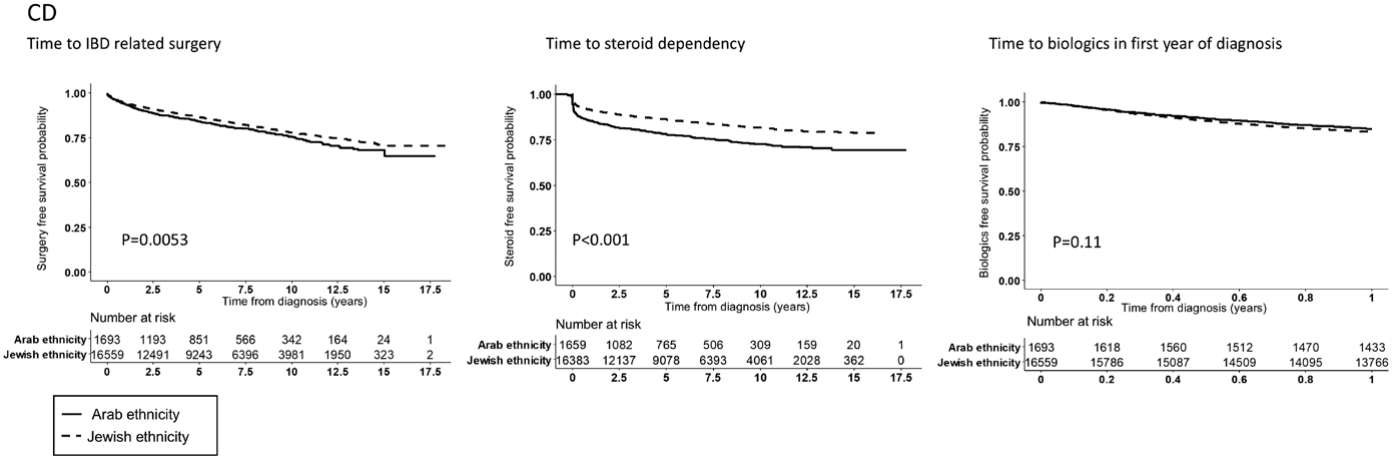
Time to Steroid dependency, time to biologics during the first year from diagnosis and time to IBD-related surgery by ethnicity in patients with CD.

**Figure 2.**
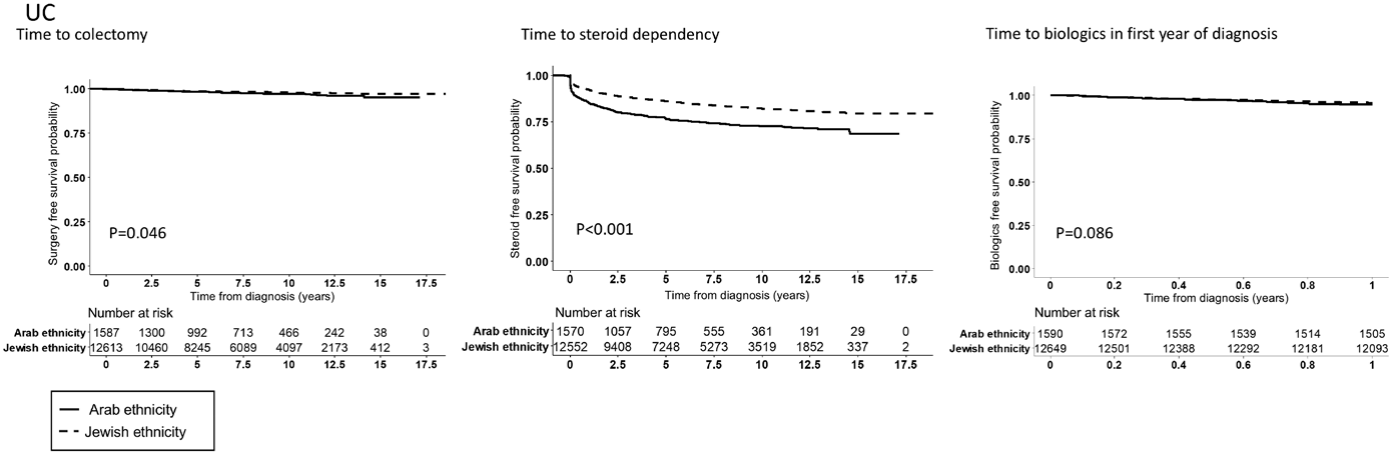
Time to Steroid dependency, time to biologics during the first year from diagnosis and time to IBD-related surgery by ethnicity in patients with UC.

Cox proportional-hazards models were used to investigate the association between ethnicity and time to disease-related outcomes adjusted for possible confounders as outlined above (Table 2). The probability of steroid dependency during the first 1-, 3-, and 5 years following diagnosis was higher in the Arab compared to the Jewish population (in CD: 17% vs. 10%, 21% vs. 14%, 23% vs. 15%; *P* < .0001 and in UC: 18% vs. 10%, 22% vs. 13% and 25% vs. 15%; *P* < .0001, respectively; Figures 1 and 2). Similarly, at 3 and 5 years, the probability of IBD-related surgery was higher in the Arab population in CD (13% vs. 10%, 16% vs. 14%, respectively; *P* = .005(, but not in UC (2% vs. 1% and 2% vs. 2 %, respectively; *P* = .06). In CD, the probability of IBD-related perianal surgery at 3 and 5 years was also higher among Arabs compared to Jews (4% vs. 3% and 6% vs. 4%, respectively, *P* < .001) (Supplementary Figure 1).

**Table 2.** IBD outcomes by ethnicity.

	Arabs	Jews	*P*-value
**Steroid dependency** Crohn’s disease At 1 year following diagnosis At 3 years following diagnosis At 5 years following diagnosis Ulcerative colitis At 1 year following diagnosis At 3 years following diagnosis At 5 years following diagnosis	246 (15%) 309 (18%) 343 (20%) 236 (15%) 313 (20%) 346 (22%)	1428 (8.6%) 1876 (11%) 2126 (13%) 996 (7.9%) 1423 (11%) 1636 (13%)	<.001 <.001
**Biologics’ initiation** ^a^ Crohn’s disease Ulcerative colitis	261 (15%) 85 (5.3%)	2800 (17%) 556 (4.4%)	.13 .1
**IBD-related admissions at 1 year of follow-up period** Crohn’s disease No hospitalizations 1 hospitalization ≥2 hospitalizations Ulcerative colitis No hospitalizations 1 hospitalization ≥2 hospitalizations	1080 (64%) 293 (17%) 320 (19%) 1191 (75%) 221 (14%) 178 (11%)	12 394 (75%) 2211 (13%) 1954 (12%) 10 601(84%) 1275 (10%) 773 (6%)	<.001 <.001
**IBD-related admissions at 3 years of follow-up period** Crohn’s disease No hospitalizations 1 hospitalization ≥2 hospitalizations Ulcerative colitis No hospitalizations 1 hospitalization ≥2 hospitalizations	936 (55%) 291 (17%) 466 (28%) 1051 (66%) 235 (15%) 304 (19%)	10 886 (66%) 2547 (15%) 3126 (19%) 9673 (76%) 1590 (13%) 1386 (11%)	<.001 <.001
**IBD-related admissions at 5 years of follow-up period** Crohn’s disease No hospitalizations 1 hospitalization ≥2 hospitalizations Ulcerative colitis No hospitalizations 1 hospitalization ≥2 hospitalizations	874 (52%) 270 (16%) 549 (32%) 965 (61%) 250 (16%) 375 (24%)	10 120 (61%) 2542 (15%) 3897 (24%) 9135(72%) 1725 (14%) 1789 (14%)	<.001 <.001
**IBD-related abdominal surgery during follow-up period** Crohn’s disease At 1 year following diagnosis At 3 years following diagnosis At 5 years following diagnosis Ulcerative colitis At 1 year following diagnosis At 3 years following diagnosis At 5 years following diagnosis	110 (6.5%) 198 (12%) 235 (14%) \9 (0.6%) 19 (1.2%) 24 (1.5%)	934 (5.6%) 1583 (9.6%) 2003 (12%) 83 (0.7%) 135 (1.1%) 170 (1.3%)	.16 .005 .036 .8 .7 .7
**IBD-related perianal surgery during follow-up period** Crohn’s disease At 1 year following diagnosis At 3 years following diagnosis At 5 years following diagnosis	36 (2.1%) 68 (4%) 83 (4.9%)	255 (1.5%) 448 (2.7%) 572 (3.5%)	.08 .003 .003

^a^During the first year from diagnosis.

Abbreviation: IBD, inflammatory bowel disease.

Reflecting utilization of inpatient health care services, Arabs had more frequent IBD-related hospitalizations than Jews during the first 1-, 3-, and 5 years following diagnosis (in CD: 19% vs. 12%, 28% vs. 19% and 32% vs. 24% with 2 or more IBD-related hospitalizations; respectively, *P* < .001 and in UC: 11% vs. 6%, 19% vs. 11% and 24% vs.14%, respectively; *P* < .001) (Supplementary Figure 2).

### Multivariable analysis

**Figure 3.**
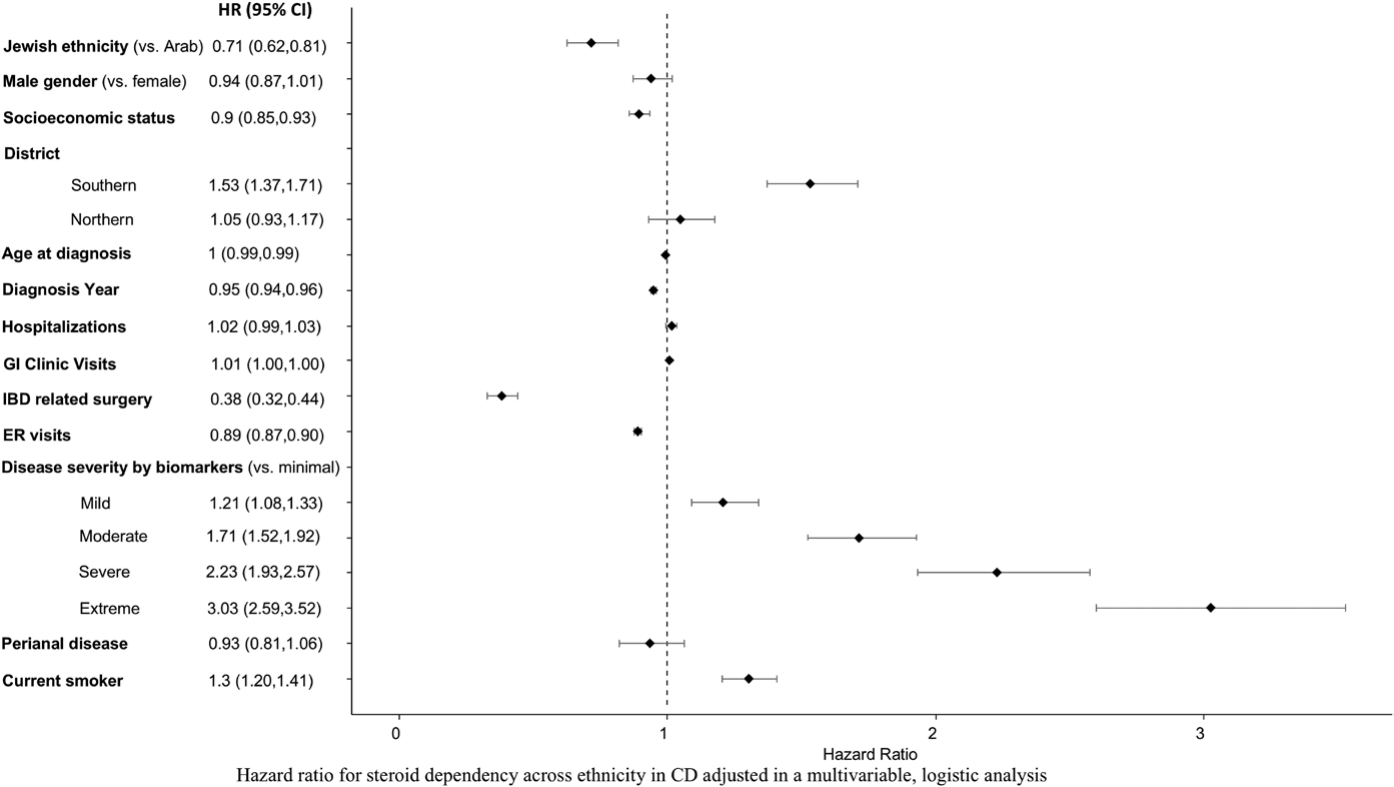
Hazard ratio for steroid dependency across ethnicity in CD adjusted in a multivariable, logistic analysis.

**Figure 4.**
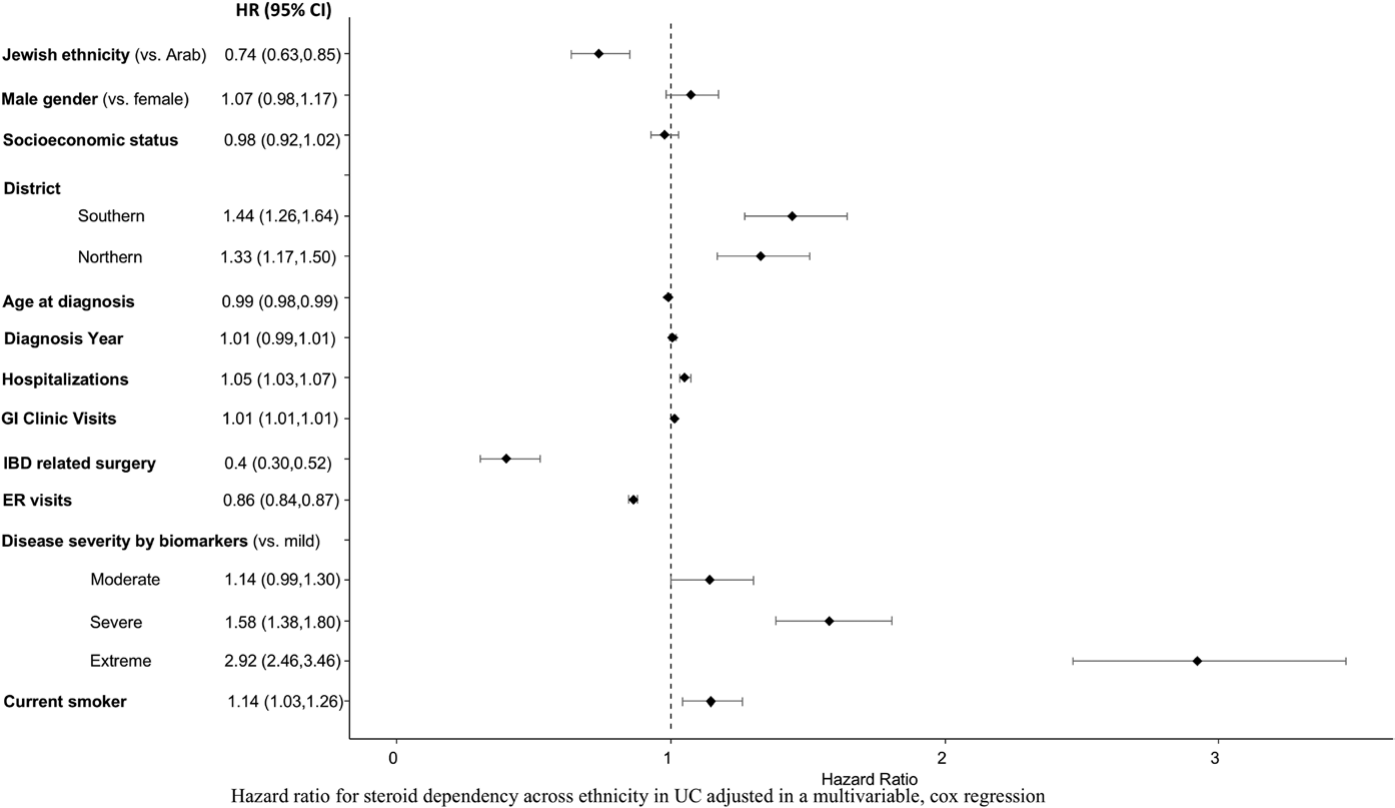
Hazard ratio for steroid dependency across ethnicity in UC adjusted in a multivariable, logistic analysis. Abbreviations: CD, Crohn’s disease; UC, ulcerative colitis.

In order to explore the relative impact of ethnicity we performed a multivariable analysis, adjusted for demographic and clinical variables (Figures 3 and 4). In the multivariate model, Jewish ethnicity was associated with 30% less steroid dependency compared to Arabs in both CD (HR = 0.7 [95% CI, 0.6-0.8]) and UC (HR = 0.7 [0.6-0.8]). Conversely, Jewish ethnicity was associated with 30% higher likelihood of using biologics during the first year from diagnosis in patients with CD (HR = 1.3 [95% CI, 1.1-1.6]) but not in UC (HR = 1.0 [95% CI, 0.8-1.4]). There was no indication of effect modification for SES and ethnicity, meaning that higher SES did not mitigate the negative impact of Arab ethnicity on disease outcome (data not shown).

## Discussion

This nationwide study provides a unique perspective on IBD care disparities between the Jewish and Arab populations in Israel. Our findings suggest that ethnicity may impact disease outcomes even when correcting for SES. Arab ethnicity was associated with a higher rate of steroid dependency, a known marker of suboptimal disease management,^18^ likely associated with the decreased use of biologics in Arab patients. Arabs also had higher utilization of inpatient health services. Our findings are consistent with those of previous selected and small cohorts showing that Arab IBD patients may have more exacerbations and hospitalizations than Jewish patients and are more likely to receive oral steroids, 5-aminosalicylic acid or immunomodulatory drugs and are less likely to receive biologics.^9,14,15^

Time to perianal surgery was also shorter in Arabs with CD, but we cannot exclude the possibility that this is associated, at least in part, with ethnic-specific differences in the perianal disease rate. Indeed, perianal disease is closely associated with ethnicity, reaching, for instance, over 50% in South Korea.^20^

Interestingly, in this study, higher SES did not minimize the negative impact of Arab ethnicity on disease outcomes. It should be noted that Israel has a universal medical insurance program with compulsory membership of one of 4 HMOs, hence ethnicity does not impact basic insurance coverage (apart from purchased supplementary private medical insurance). Therefore, Israel is a good example to explore ethnic deviation of care by eliminating the factor of medical insurance availability. One potential explanation for the observed gap in IBD management is likely related to limited availability of specialized healthcare services rather than the cost of treatment. Indeed, patients treated in high-volume IBD centers have more favorable disease outcome than those treated in small centers,^21,22^ as do hospitalized patients managed by a gastroenterologist compared to an internist^23^ and those receiving care provided by gastroenterologists with a specific interest in IBD compared to those without.^24^ Delay in diagnosis, in part due to poor access to gastroenterologists, has also been shown to negatively impact disease course.^25,26^ Additional factors potentially contributing to suboptimal management among the Arab population may include unmeasured psychosocial and environmental influences. These could encompass differing attitudes toward and adoption of nutritional IBD interventions across ethnic groups,^27^ as well as cultural barriers and healthcare systems biases, as highlighted by Daoud et al.^28^

In a previous report from Israel, Mahamid et al.^15^ showed a substantially higher smoking rate among Arab IBD patients compared with Jewish patients (46% vs. 7%). In our study, smoking was indeed more frequent among Arabs but only in CD patients, while in patients with UC smoking was more prevalent in Jews. This could serve as another explanation for the poorer disease course since smoking worsens CD and protects, or at least does not affect, UC outcomes.^29,30^ However, smoking and the other possible confounders (eg, SES, residence in peripheral regions and age) were accounted for in the multivariable model and the correlation between ethnicity and poorer outcomes persisted. As an interesting side finding of the multivariate model smoking was associated with an increased risk for steroid dependency not only in CD (HR = 1.3 [1.2-1.5]) but also in UC (HR = 1.1[1.05-1.3]).

Several studies have described adverse healthcare outcomes among ethnic groups in various chronic diseases such as end-stage kidney disease and multiple sclerosis.^1,2^ Differences in socioeconomic, cultural, immigration, and genetic factors, as well as conscious or subconscious racial bias of health care providers may underlie such findings.^7,8,31,32^ Prior studies examining the impact of low income on healthcare delivery in IBD have shown conflicting results. Lower SES has been previously associated with a deleterious impact on IBD outcomes.^8^ Nahon et al.^32^ reported no differences in corticosteroid dependency, immunosuppressive or biologic therapy between socioeconomically “deprived” vs “non-deprived” patients, but more frequent hospitalizations and higher rate of surgical intervention in the latter. Conversely, Santos et al.,^7^ found that Indigenous populations in Canada, despite being part of a universal healthcare system, had higher rates of hospital admissions and fewer prescription medication claims for IBD, with no observed differences in gastroenterology visits or surgical rates.

A distinct advantage of our study is the nationwide cohort that represents the entire population without selection bias and with accurate data relating to drug prescriptions and procedures, cross-referenced to the prospective Ministry of Health databases. However, there are several limitations to our study. First, it can only suggest associations between variables, not causality. It is possible that despite the multivariable adjustment, specific unknown confounding variables could explain some of the association between ethnicity and outcomes. Another limitation, inherent to administrative-based studies, is that we were restricted to the available data in the electronic records from the HMOs and some valuable data were missing or were not valid, such as disease extent, endoscopic surveillance, and smoking.

In summary, we found that Arab ethnicity is associated with adverse IBD management outcomes. In the setting of comparable health care services via HMO, these differences, which may involve racial, socioeconomic, geographical, cultural, and genetic factors, highlight the need for population-focused interventions such as promoting equitable access to biologics and addressing potential cultural and systemic barriers in healthcare delivery in order to improve access to medical care and maximize IBD outcomes across all populations.

## Supplementary Material

otaf025_suppl_Supplementary_Data

## Data Availability

Data, analytical methods, and study materials will be made available on request to the corresponding author.
