# Supplementary material for: Ethnic Disparities in the Management of Inflammatory Bowel Disease in Israel and Impact on Outcomes
Source: Crohns Colitis 360. 2025 Mar 31;7(2):otaf025. doi: 10.1093/crocol/otaf025 (PMC12048840; doi:10.1093/crocol/otaf025)
Supplement: otaf025_suppl_Supplementary_Data [file otaf025_suppl_supplementary_data.zip › Legends_Supp.docx]

**LEGENDS to SUPPLEMENTARY FIGURES**

- **Suppl Figure 1:** Time to perianal surgery by ethnicity in patients with CD
- **Suppl Figure 2:** IBD-related hospitalizations at 1, 3, and 5 years of follow up by ethnicity in CD and UC
