# Supplementary figures and images for: Ethnic Disparities in the Management of Inflammatory Bowel Disease in Israel and Impact on Outcomes
Source: Crohns Colitis 360. 2025 Mar 31;7(2):otaf025. doi: 10.1093/crocol/otaf025 (PMC12048840; doi:10.1093/crocol/otaf025)

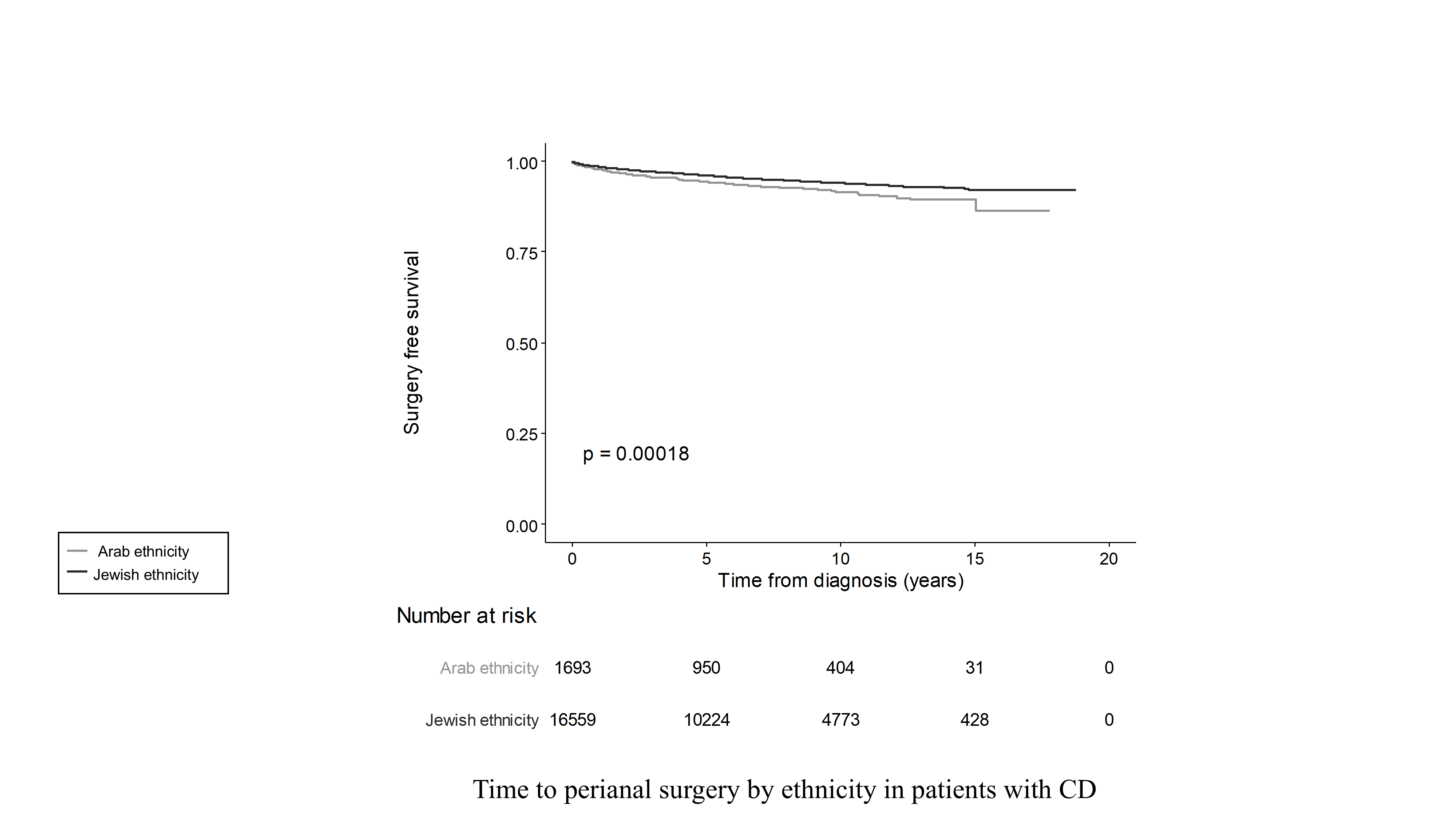

Supplement: otaf025_suppl_Supplementary_Data [file otaf025_suppl_supplementary_data.zip › SuppFigure1.tif]

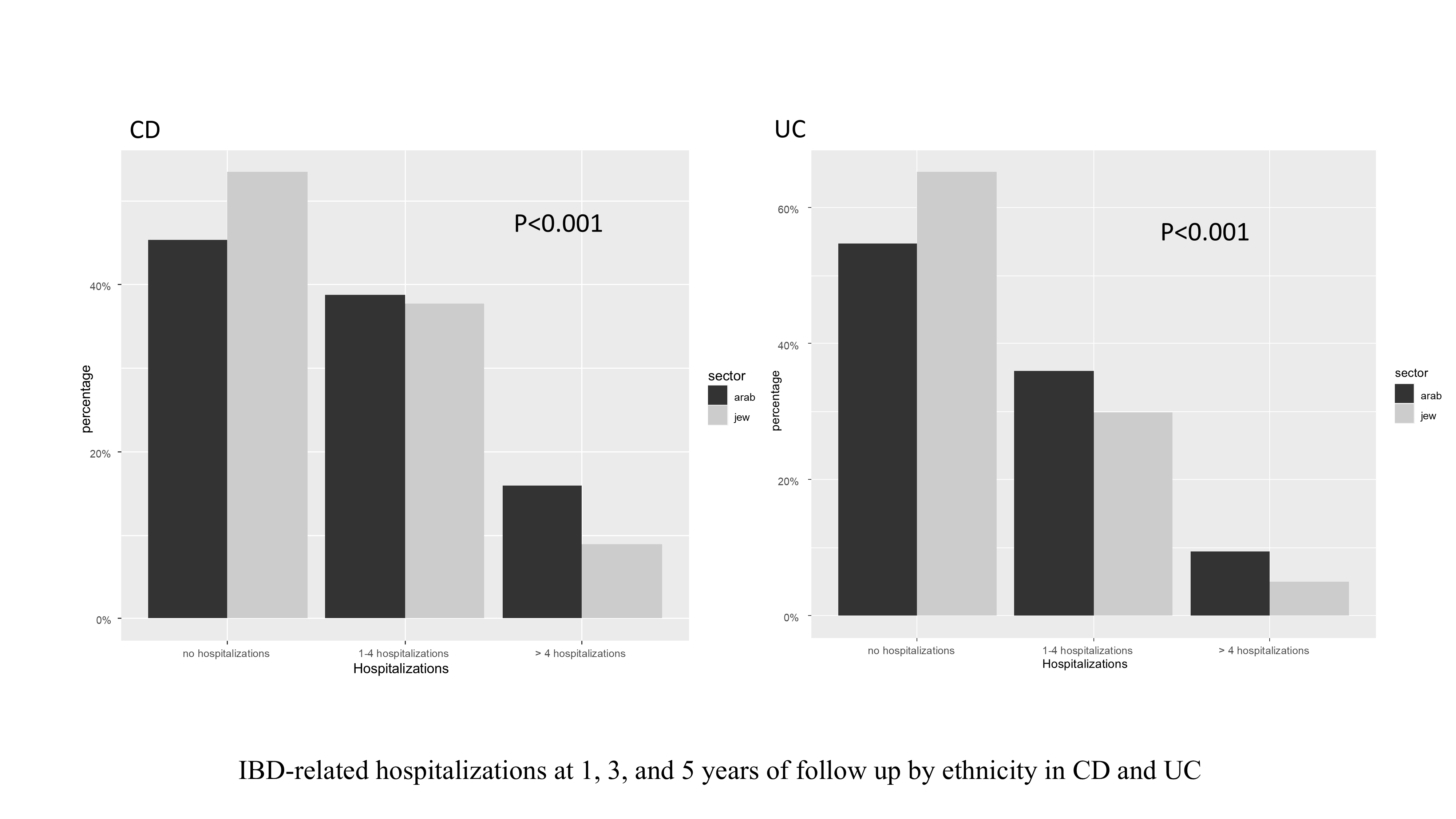

Supplement: otaf025_suppl_Supplementary_Data [file otaf025_suppl_supplementary_data.zip › SuppFigure2.tif]
